# Supplementary figures and images for: Exposure to small molecule cocktails allows induction of neural crest lineage cells from human adipose-derived mesenchymal stem cells
Source: PLoS One. 2020 Oct 26;15(10):e0241125. doi: 10.1371/journal.pone.0241125 (PMC7588063; doi:10.1371/journal.pone.0241125)

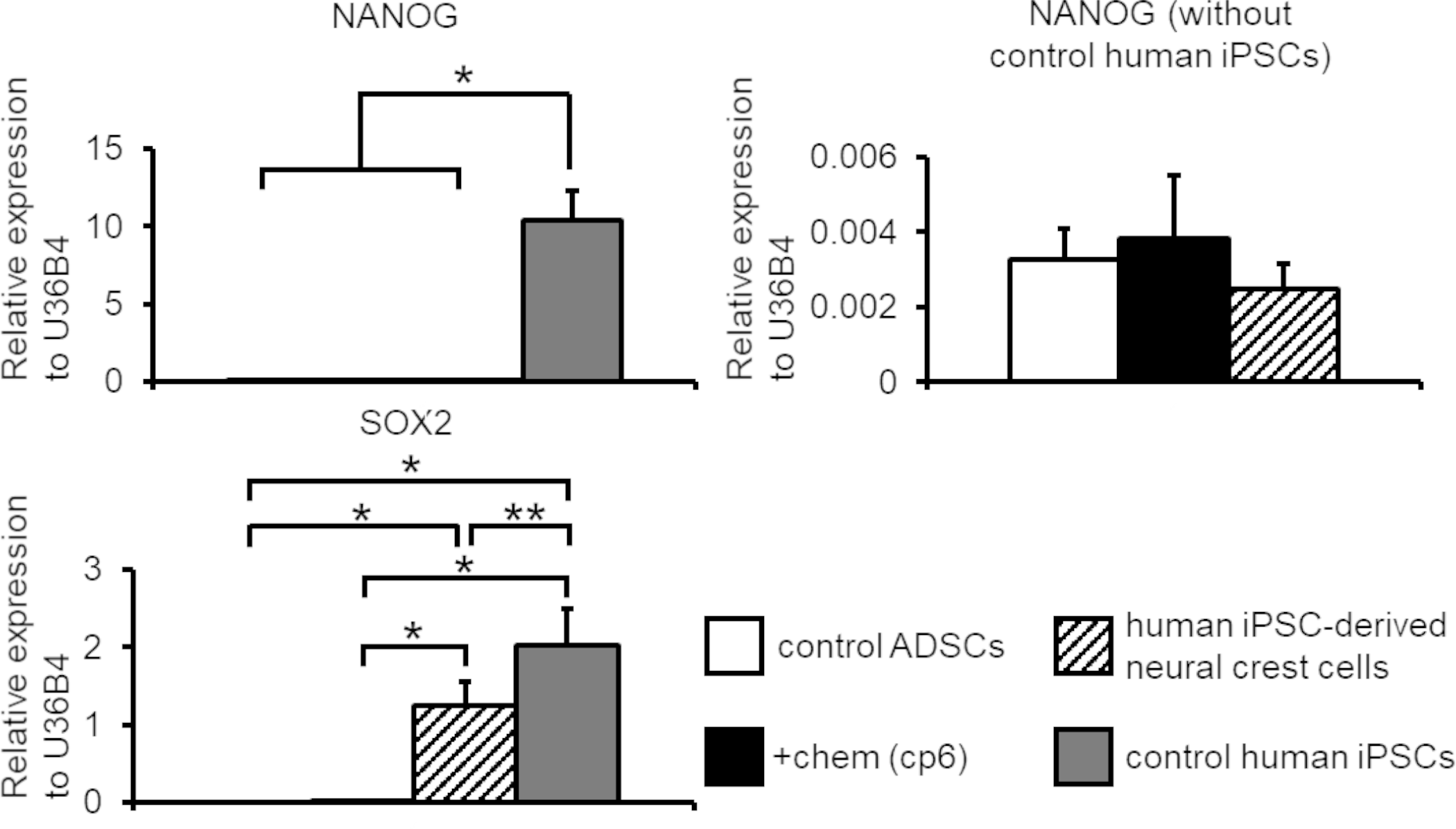

Supplement: S1 Fig — The expression values were normalized to that of U36B4. (n = 3, error bar shows SDs; one-way ANOVA Tukey post-test). (TIF) [file pone.0241125.s002.tif]

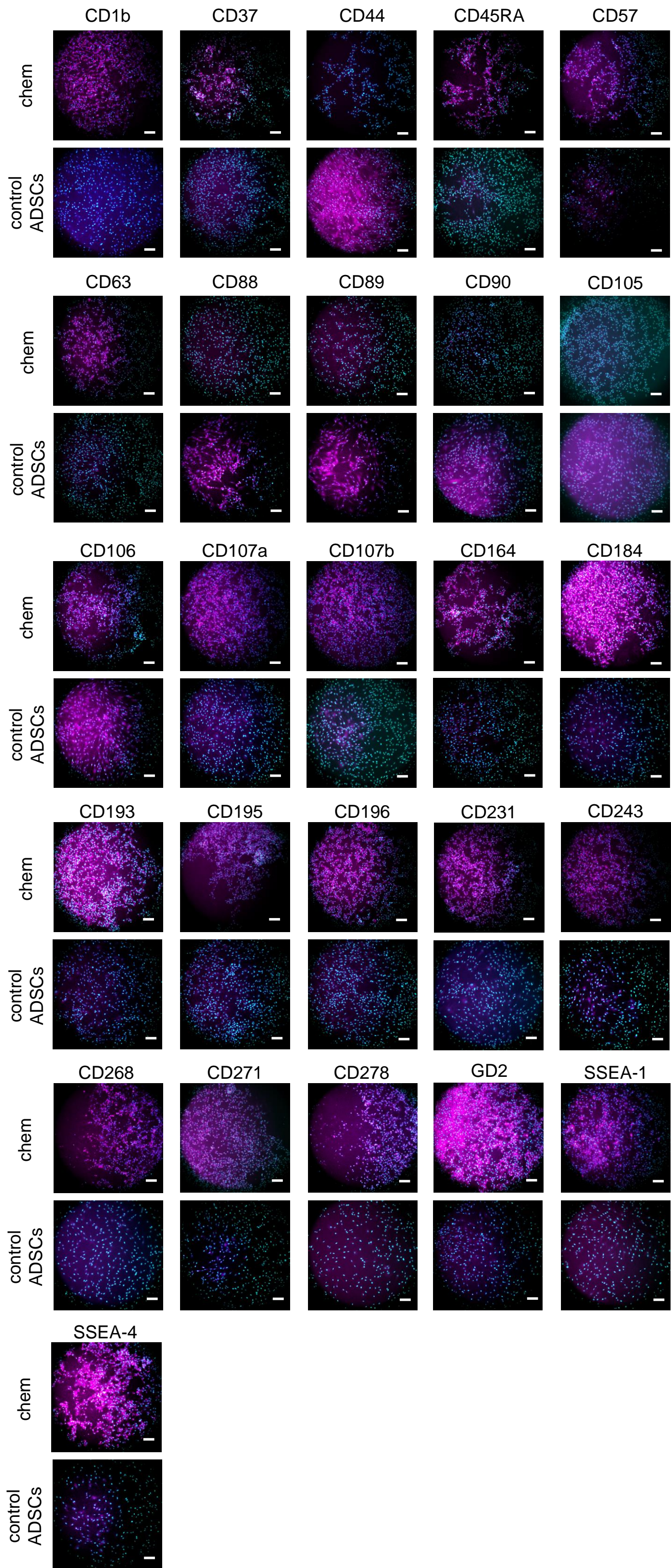

Supplement: S2 Fig — (PDF) [file pone.0241125.s003.pdf]
